# Lab-on-Fiber Nanoprobe with Dual High-Q Rayleigh Anomaly-Surface Plasmon Polariton Resonances for Multiparameter Sensing

**DOI:** 10.1038/s41598-018-38113-1

**Published:** 2019-02-13

**Authors:** Hyun-Tae Kim, Miao Yu

**Affiliations:** Department of Mechanical Engineering, University of Maryland, College Park, Maryland 20742 USA

## Abstract

Surface plasmon resonance (SPR) based sensing is an attractive approach for realizing lab-on-fiber nanoprobes. However, simultaneous measurement of multiple parameters (e.g., refractive index and temperature) with SPR-based nanoprobes, although highly desirable, is challenging. We report a lab-on-fiber nanoprobe with dual high-Q Rayleigh anomaly (RA)-surface plasmon polariton (SPP) resonances for multiparameter sensing. To achieve high-Q RA-SPP resonance the nanoprobe employs a plasmonic crystal cavity enhanced by distributed Bragg reflector (DBR) gratings on the end-face of a single-mode optical fiber. By tailoring the grating periods of the plasmonic crystal cavity and DBRs, two spatially separated high-Q RA-SPP resonance modes are designed within a 50 nm spectral range in C + L band. The fabricated nanoprobe demonstrates two RA-SPP resonances near 1550 nm with high Q-factors up to 198. These two high-Q resonances are further showed to exhibit distinctive responses to the changes of refractive index and temperature, which enables simultaneous measurements of both parameters. The proposed lab-on-fiber nanoprobes will pave the way for realizing compact multiparameter sensing solutions compatible with optical communication infrastructures.

## Introduction

Lab-on-fiber technology^1–4^ offers great potential for sensing in harsh environments (e.g., high temperature, corrosive, and high electromagnetic interference environments) and hardly accessible areas (e.g., inside human body, complex pipeline, and remote terrestrials). Most lab-on-fiber probes have the photonic sensing structures built on a fiber end-face where light can effectively interacts with measurands^5^.

Various photonic sensing structures have been employed for fiber end-face sensors, including Fabry-Perot cavities^6,7^, photonic crystals^8–11^, metamaterials^12–14^, and plasmonic nanostructures^15–18^. Among them on-fiber surface plasmon resonance (SPR) sensors have received increasing attention due to their compact sizes and superior sensitivities.

SPR sensors utilize surface plasmon polaritons (SPPs) that can be excited through an array of subwavelength metallic nanostructures (e.g., holes, patches, and gratings)^19–22^. These nanostructures help provide incident photons additional momentum for coupling into SPPs. Light diffraction from these grating structures can often lead to another optical phenomenon called Rayleigh anomaly (RA). SPPs and RAs interact under certain conditions, which results in strong and sharp RA-SPP resonances^23,24^. SPR sensors can greatly benefit from these high-Q resonances for achieving better accuracy.

Most on-fiber SPR sensors are based on the measurement of refractive index change induced resonance wavelength shift, which is highly sensitive to the surface characteristics. Therefore, by applying a functional layer (e.g., metal-organic frameworks, ligands, and polymer) on the metallic surface, various parameters such as concentration of chemical and biological gases and fluids, biomolecules, and dynamic pressure can be measured^25–27^. On-fiber SPR sensors often suffer from low Q-factors due to their small mode field areas limited by the fiber core sizes. SPR sensors based on single-mode optical fibers face more challenges. Recently, high-Q SPR sensors were demonstrated on single-mode optical fibers by using a plasmonic crystal cavity^28,29^ and a distributed-feedback surface plasmon resonator^30^ for biochemical sensing and ultrasound sensing.

It is noted that most of the on-fiber SPR sensors reported in the literature were designed for single parameter measurements. However, a major limitation of the SPR sensors designed solely for refractive index measurement is the degradation of sensing accuracy due to temperature effect. For example, in chemical and biological sensing, the refractive indices of some chemicals (e.g., hydrochloric acid and sulfuric acid) and biological solutions (e.g., glucose and protein) change with the solution concentration as well as the solution temperature. To ensure sensing accuracy, temperature compensation with an additional sensor is often required, which increases the complexity of the sensing system. An attractive alternative is an on-fiber multiparameter sensor, which supports multiple SPRs with distinctive responses to the changes of refractive index and temperature. However, it is challenging to obtain multiple high-Q SPRs that possess distinctive responses to different parameters within the limited spectral range of a light source.

Previously, our group reported a multiparameter sensor that utilized the combined response from a single SPR and a plasmonic interferometer obtained with an Au nanohole array fabricated on a single-mode optical fiber^31^. However, the sensor required rather complicated post signal processing to obtain the sensor responses from the SPR wavelength shift and the optical path difference change of the interferometer. Furthermore, the small mode area and broad range of incident angles of the fiber guided mode resulted in a relatively low-Q (~50) SPR, which limited the accuracy of the SPR wavelength measurement.

**Figure 1:**
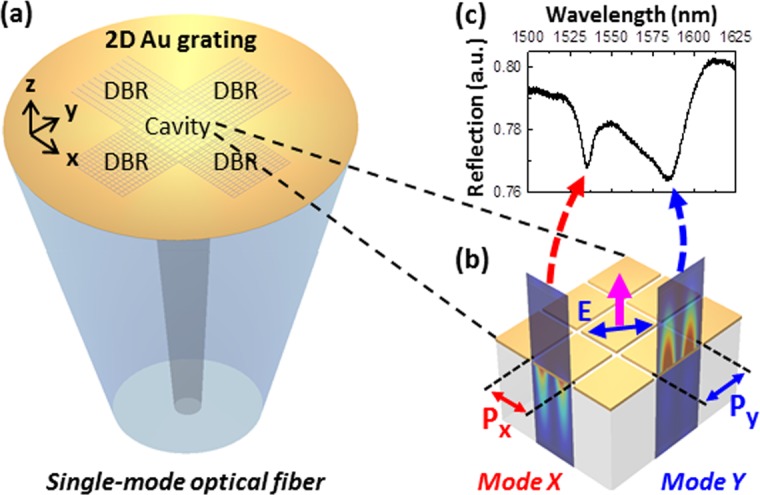
Schematics of (**a**) the lab-on-fiber multiparameter nanoprobe with a plasmonic crystal cavity on the fiber end-face and (**b**) two spatially separated RA-SPP resonance modes excited in the plasmonic crystal cavity. (**c**) The reflection spectrum from the nanoprobe with two resonances near 1550 nm.

Here, we report an SPR-based lab-on-fiber multiparameter nanoprobe for simultaneous refractive index and temperature measurements. The nanoprobe employs a plasmonic crystal cavity on the end-face of a single-mode optical fiber (Fig. 1a). The plasmonic crystal cavity consists of a two-dimensional (2D) rectangular Au grating in the core surrounded by distributed Bragg reflector (DBR) gratings in the cladding area. The plasmonic crystal cavity with DBRs supports dual high-Q RA-SPP resonances near 1550 nm, which are designed to be spatially separated: one in the solution medium and another in the silica medium (Fig. 1b). Owing to the spatial separation of the two resonance modes, the ambient refractive index change affects mainly the response of the mode in the solution medium; while the temperature change affects the responses of both modes differently based on the thermo-optic coefficients of the mediums. Therefore, the responses of the mode in the solution medium to the changes of ambient refractive index and temperature become distinctively different from those of the mode in the silica medium, which enables the measurement of both parameters simultaneously. The DBRs are designed to enhance the Q-factor of the RA-SPP resonances of the single-mode fiber device. Furthermore, the two resonance wavelengths are designed to be within a 50-nm spectral span near 1550 nm (Fig. 1c), so that the nanoprobe can be compatible with the C + L band optical communication infrastructures.

## Results and Discussion

Our on-fiber plasmonic crystal design exploits a thin 2D rectangular subwavelength Au grating (thickness: 60 nm, slit width: 50 nm). When a transverse-magnetic (TM) plane wave is incident on the Au grating with a period P, the incident photons gain additional momentum in integer multiples of G = 2π/P, which excite SPPs at the Bragg coupling condition^32,33^:

1 $$\mathrm{Re}(\frac{\omega }{c}\sqrt{\frac{{\varepsilon }_{Au}{\varepsilon }_{d}}{{\varepsilon }_{Au}+{\varepsilon }_{d}}})=|{k}_{0}\,\sin \,\theta \pm iG|.$$

**Figure 2:**
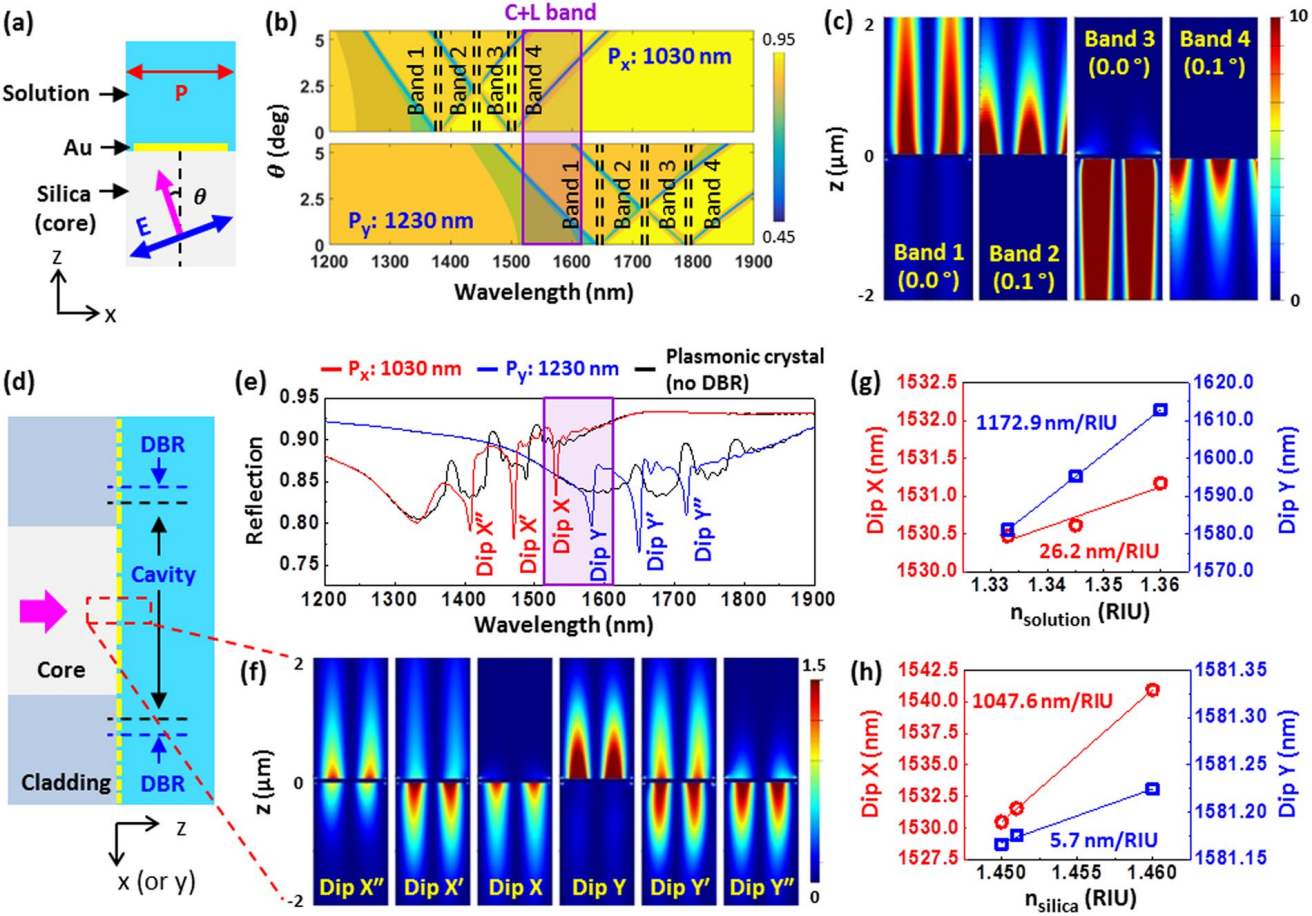
(**a**) A unit cell of the Au grating with an incident TM plane wave. (**b**) Reflection spectra of infinite 1D Au gratings with P_x_ = 1030 nm and P_y_ = 1230 nm at different incident angles obtained with 2D FDTD simulations. (**c**) Simulated band edge mode profiles for the Au grating of 1230 nm period at near normal incident angles. (**d**) On-fiber plasmonic crystal cavity with a fiber-guided mode. (**e**) Simulated reflection spectra of the 1D on-fiber plasmonic crystal cavities with 1030-nm (red curve) and 1230-nm (blue curve) periods of core gratings compared with the corresponding pure plasmonic crystals on the entire fiber-face without DBR gratings (black curves), respectively. (**f**) Mode distribution profiles corresponding to different resonance dips in (**e**). (**g**) Dip X and Dip Y wavelengths as a function of simulated refractive index change of the solution. (**h**) Dip X and Dip Y wavelengths as a function of simulated refractive index change of the silica (i.e., fiber core).

Here ω, c, and k_0_ are the angular frequency, speed, and momentum of free-space light. ε_Au_ and ε_d_ are the relative permittivities of Au and adjacent dielectric materials (i.e., ambient solution and silica), respectively. θ is the incident angle. Integer i denotes specific SPP modes. On the other hand, RAs are excited by the diffracted waves parallel to the grating surface at the condition of

2 $$\mathrm{Re}(\frac{\omega }{c}\sqrt{{\varepsilon }_{d}})=|{k}_{0}\,\sin \,\theta \pm jG|,$$

where j is the integer denoting the diffraction orders. The interplay between the SPPs and RAs renders sharp resonance dips in its reflection spectrum^23^. For the on-fiber gratings, the incident angles are determined by the numerical aperture (NA) of the optical fiber. Therefore, by tailoring the x- and y-directional periods (P_x_ and P_y_) of the 2D rectangular Au grating the spatially separated RA-SPP resonances can be engineered and positioned in the desired spectral range. Based on 2D finite-difference time-domain (FDTD) simulations of a unit cell Au grating (1D grating) (Fig. 2a), P_x_ and P_y_ are chosen to be 1030 nm and 1230 nm respectively to support the spatially separated RA-SPP resonances in the C + L band (i.e., 1530–1625 nm). Figure 2b shows the reflection spectra of the unit cell Au gratings with periods of 1030 nm and 1230 nm, respectively, at different incident angles that are supported by a single-mode optical fiber (NA: 0.13). For both 1D gratings, the RA-SPP resonances diverge into a short and long-wavelength branches with the increased incident angle and they are grouped into four different resonance bands. Band 1 of the 1230-nm period grating and Band 4 of the 1030-nm period grating are positioned in the C + L band. Figure 2c shows the band edge mode profiles (|E_z_|^2^) of the 1230-nm period grating at near normal incident angles. The modes at the edges of Band 1 and Band 2 are distributed in the solution side and represent RAs and SPPs, respectively. On the other hand, the modes at the edges of Band 3 and Band 4 are distributed in the silica side, representing RAs and SPPs, respectively. Note that the 1030-nm period grating has similar mode profiles to the 1230-nm period grating (data not shown). Given the band structure profiles of the two 1D gratings (Fig. 2b), for a 2D rectangular Au grating with P_x_ of 1030 nm and P_y_ of 1230 nm, it is conceivable that there exists two resonance modes that are spatially separated in the C + L band: Band 1 (from P_y_ = 1230 nm) in the solution side and Band 4 (from P_x_ = 1030 nm) in the silica side. As a validation, 3D FDTD simulations are performed for a unit cell of the 2D rectangular Au grating (see Fig. S1a). The simulated reflection spectra of the 2D rectangular Au grating confirm that two spatially separated resonance modes are excited in the C + L band (Fig. S1b,c).

To obtain deeper and sharper resonance dips (i.e., higher Q-factors) of the excited RA-SPPs, we exploit surrounding DBR gratings (Fig. 1a) to form a 2D plasmonic crystal cavity. The band gaps of the DBRs are designed to confine the two RA-SPP resonance modes in the C + L band. Small gaps are placed between the DBRs and the plasmonic crystals to enable constructive interference between the reflections from the DBR gratings and cavity edge^28^. 2D FDTD simulations are carried out to guide the design of a 1D on-fiber plasmonic crystal cavity illuminated with a fiber-guided mode (Fig. 2d). To position the resonances at ~1530 nm for the silica mode and ~1575 nm for the solution mode, the DBR gratings are designed to have a 525-nm period with a 900-nm gap in the X direction and a 590-nm period with a 550-nm gap in the Y direction. Figure 2e shows the simulated reflection spectra of two 1D plasmonic crystal cavities compared with the corresponding plasmonic crystals without DBR gratings (i.e., P_x_ or P_y_ Au gratings on the entire fiber end-face). Compared with those of plasmonic crystals without DBRs, the spectra of plasmonic crystal cavities render three sharper resonances with deeper extinction and much higher Q-factors, which result from three band gaps of the DBR gratings. The band gaps in the short (Dips Xʹʹ and Y) and long (Dips X and Yʹʹ) wavelengths help confine the resonance modes in the ambient solution and silica sides, respectively; while the resonance modes corresponding to the intermediate band gaps (Dips Xʹ and Yʹ) are confined in both ambient solution and silica sides. Clearly, in the C + L band, the designed 2D plasmonic crystal cavity has two spatially separated resonance modes (Dips X and Y), which is the key to realizing simultaneous ambient solution refractive index and temperature measurements. The simulated Q-factors are 275 and 120 for Dips X and Y, respectively. Because the modes associated with Dips X and Y are spatially separated and confined in the silica and ambient solution sides, respectively (Fig. 2f), in the simulations, Dips X and Y are shown to respond selectively with a high sensitivity to the refractive index changes of the silica (sensitivity of 1047.6 nm/RIU) and the ambient solution (sensitivity of 1172.9 nm/RIU) (Fig. 2g,h), respectively. Assuming the thermo-optic coefficient of silica is 1 × 10^−5^/°C, the temperature sensitivity of Dip X is 0.0104 nm/°C. Note that both dips have weak cross-sensitivities: the sensitivities of Dips X and Y to the refractive index changes of the ambient solution and silica are 26.2 nm/RIU and 5.7 nm/RIU, respectively. These cross-sensitivities result from the weak mode fields penetrated into the opposite side through the slits.

For the resonance dips adjacent to the C + L band (i.e., Dips Xʹ and Yʹ), the modes are confined in both ambient solution and silica sides and thus have lower selectivities and sensitivities than those of Dips X and Y. For example, the sensitivities of Dip X’ to the ambient solution and the silica are almost the same (532.9 nm/RIU and 534.4 nm/RIU, respectively), which cannot be used for multiparameter sensing^8^. Dip Y′ has a similar behavior with the sensitivities to the ambient solution and the silica being 589.6 nm/RIU and 586.4 nm/RIU, respectively. On the other hand, although Dips X″ and Y″ are spatially separated, their wavelength dips are too far apart (beyond the spectral range of most light sources) and their cross-sensitivities are stronger than those of Dips X and Y. Therefore, compared with Dips X and Y, they are less desirable for multiparameter sensing.

**Figure 3:**
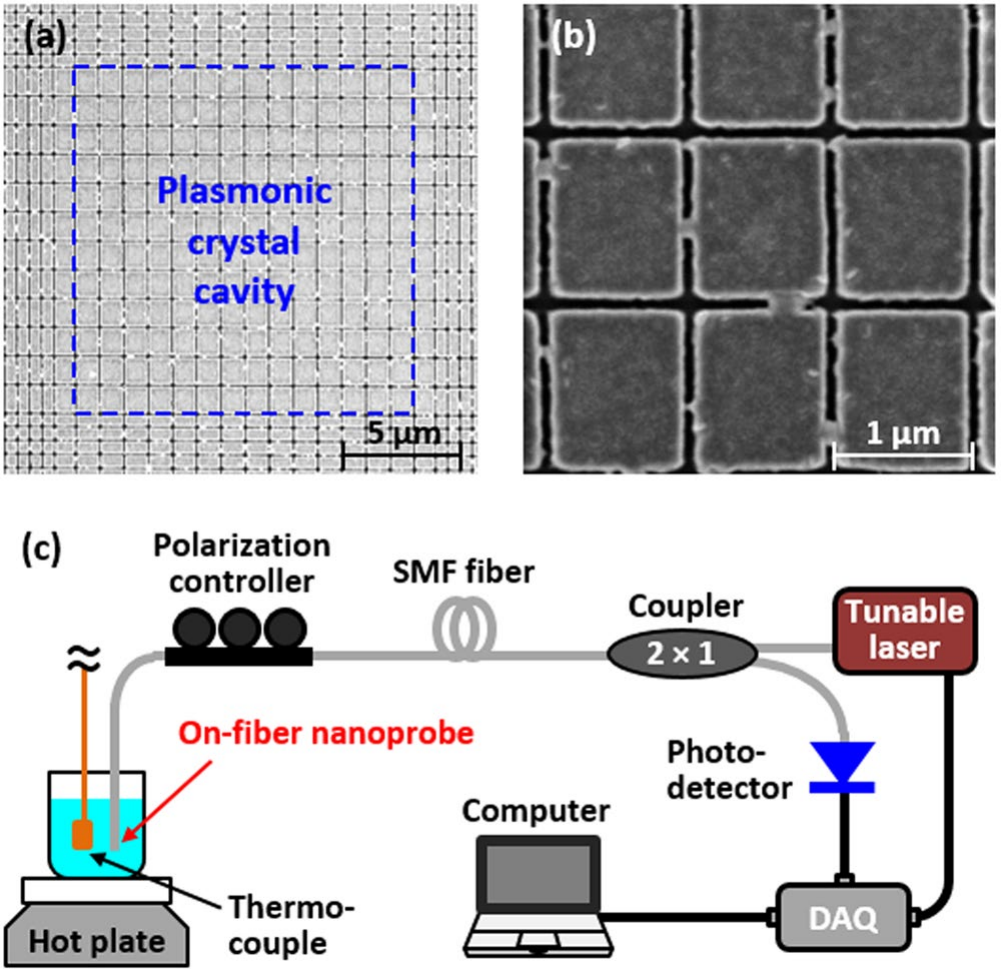
SEM images of (**a**) the plasmonic crystal cavity of the fabricated multiparameter nanoprobe and (**b**) the 2D rectangular Au grating inside the plasmonic crystal cavity. (**c**) Schematic of the experimental setup for characterization of the nanoprobes.

The designed nanoprobe was fabricated directly on the end face of a single-mode optical fiber. The plasmonic crystal cavity was created by pattering of the Au film on the fiber end-face with focused ion beam (FIB) milling. Compared with the previously reported pattern-transfer approaches using a polymer adhesive or van der Waals force^9,28,34^, the direct FIB milling method has the following advantages: i) the plasmonic crystal cavity can be accurately positioned on the tiny fiber core and ii) the strong bonding of plasmonic crystal cavity to the fiber end-face enables the nanoprobe to operate in relatively harsh environments (e.g., solvents and moderately high temperature (~100s °C) environments). The scanning electron microscope (SEM) images of the fabricated plasmonic crystal cavity on the fiber core and the rectangular Au grating inside the cavity are shown in Fig. 3a,b, respectively. The footprint of the cavity is 14.5 µm × 14.5 µm. The P_x_ and P_y_ of the core gratings are measured to be 1035 nm and 1250 nm, respectively, and the slit width is measured to be 80 nm. The Au film thickness, including a 3 nm of Ti adhesion layer, is measured to be around 68 nm. The fabricated nanoprobe is reproducible: based on three fabricated nanoprobe samples, the measured P_x_ and P_y_ of the core gratings are 1039 nm ± 7 nm and 1250 nm ± 3 nm, respectively, and the measured slit width is 81 nm ± 18 nm. Figure 3c shows the experimental setup used to characterize the fabricated nanoprobe. Reflection spectrum of the nanoprobe is obtained by using a tunable laser (TSL-510, Santec), a 2 × 1 coupler, and a photodetector (2011-FC, New Focus). The polarization state of the incident light is controlled with a polarization controller (FPC030, Thorlabs).

**Figure 4:**
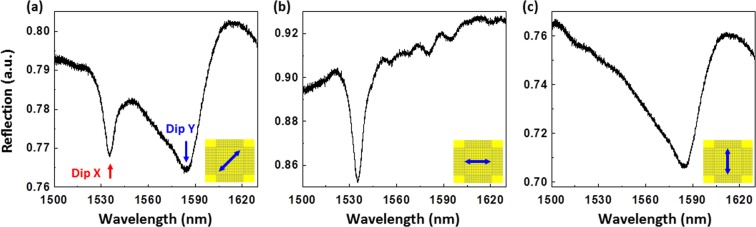
Reflection spectra obtained with the fabricated multiparameter nanoprobe at different polarizations in the room temperature DI water: (**a**) 45°-angled, (**b**) x-directional, and (**c**) y-directional linear polarizations.

Figure 4 shows the reflection spectra of the fabricated nanoprobe at different polarizations in the room temperature deionized (DI) water. Two RA-SPP resonance dips are observed at the 45°-angled linear polarization (Fig. 4a). The x- and y-directional linear polarizations render a single resonance dip in each corresponding reflection spectrum (Fig. 4b,c, respectively), which confirms the grating-period dependence of the resonance. The dips at ~1535.25 nm and ~1583.12 nm are associated to the P_x_ and P_y_ Au gratings, respectively, which agree well with the simulation results. The Q-factors of the two resonances are measured to be 198 for Dip X and 41 for Dip Y, which are lower than those obtained from the simulations (275 for Dip X and 120 for Dip Y). This discrepancy is believed to result from fabrication errors. For example, the slit width of the fabricated grating (80 nm) is wider than that used in the simulations (50 nm). According to the simulation results shown in Fig. S2, the reflection spectra of a plasmonic crystal cavity with an 80 nm slit width has lower Q-factors (268 for Dip X and 31 for Dip Y) than those obtained with a plasmonic crystal cavity with a 50 nm slit width. Moreover, the Au residual in the slits of the fabricated sample (Fig. 3b) may affect the Q-factor due to the roughened grating edges.

By using the fabricated nanoprobe with Dips X and Y, multiparameter sensing of refractive index and temperature can be performed based on a sensitivity matrix method^31,35^ as

3 $$(\begin{array}{c}n\\ T\end{array})={S}^{-1}(\begin{array}{c}{\rm{\Delta }}DipX\\ {\rm{\Delta }}DipY\end{array})+(\begin{array}{c}{n}_{0}\\ {T}_{0}\end{array}),$$

where n and T are the refractive index and temperature of the ambient solution, respectively, and n_0_ and T_0_ are the reference refractive index and temperature, respectively. S is the sensitivity matrix of the nanoprobe, which is defined as

4 $$S=(\begin{array}{c}\begin{array}{cc}{s}_{{\rm{\Delta }}n}^{X} & {s}_{{\rm{\Delta }}T}^{X}\end{array}\\ \begin{array}{cc}{s}_{{\rm{\Delta }}n}^{Y} & {s}_{{\rm{\Delta }}T}^{Y}\end{array}\end{array}).$$

Here, ${s}_{{\rm{\Delta }}n}^{X}$ and ${s}_{{\rm{\Delta }}n}^{Y}$ are the sensitivities of Dips X and Y to the refractive index change of the ambient solution at the reference temperature T_0_, respectively, and ${s}_{{\rm{\Delta }}T}^{X}$ and ${s}_{{\rm{\Delta }}T}^{Y}$ are the sensitivities of Dips X and Y to the ambient temperature change at the reference ambient solution refractive index n_0_, respectively. It is required that the ${s}_{{\rm{\Delta }}T}^{X}$ and ${s}_{{\rm{\Delta }}T}^{Y}$ are solely determined by the thermo-optic effect of silica (i.e., optical fiber). With this method, the refractive index and temperature of any unknown solution can be measured simultaneously.

**Figure 5:**
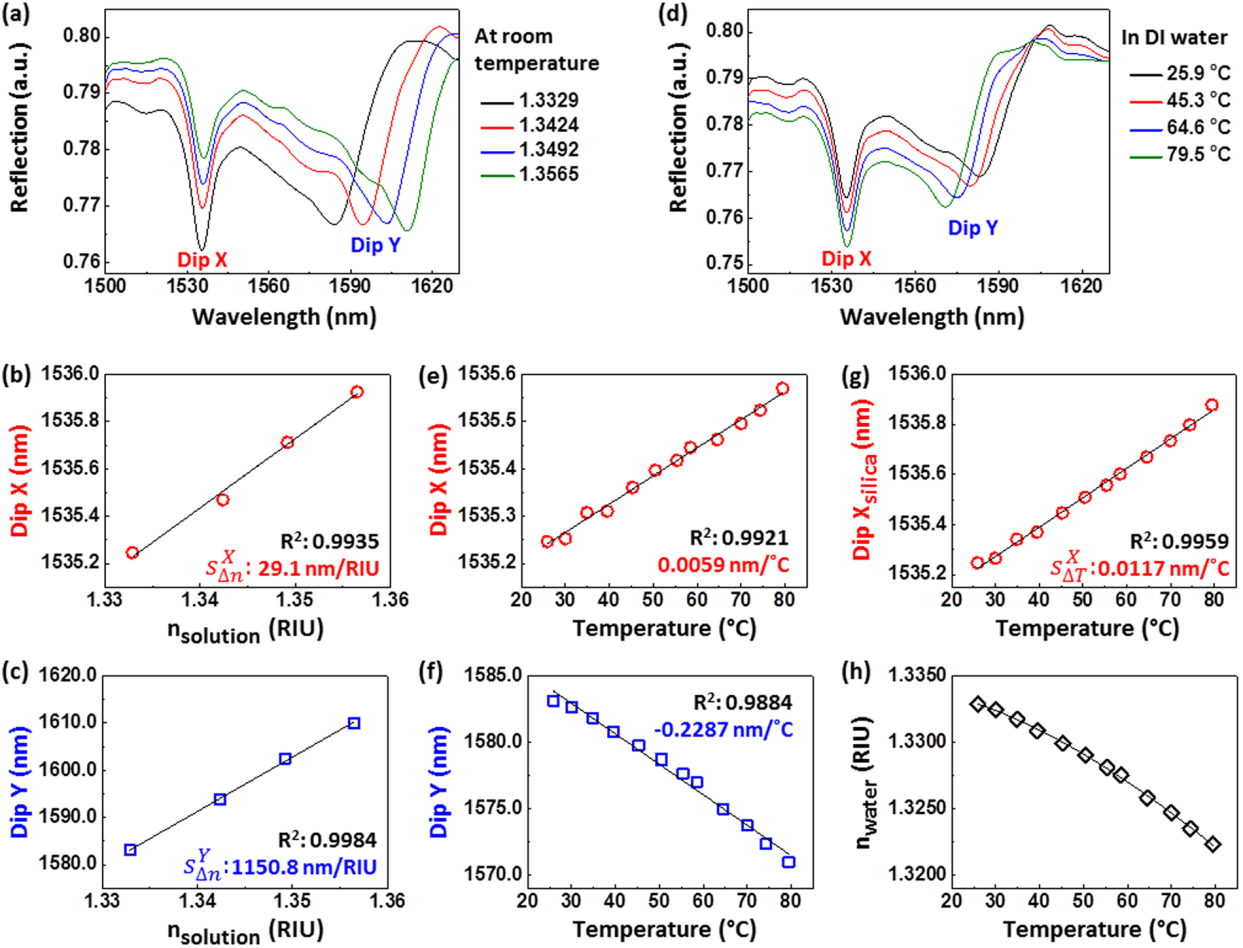
(**a**) Reflection spectra of the fabricated nanoprobe obtained for different reflective indices of glucose solutions at room temperature. Measured (**b**) Dip X and (**c**) Dip Y wavelengths as a function of refractive index of the glucose solution. (**d**) Reflection spectra of the fabricated nanoprobe obtained for different temperatures of DI water. Measured (**e**) Dip X and (**f**) Dip Y wavelengths as a function of DI water temperature. (**g**) Temperature-dependent Dip X shifts by the thermo-optic effect of silica only. (**h**) Refractive index of DI water as a function of temperature obtained from (**c**,**f**).

To obtain the four sensitivities required in the sensitivity matrix S, we characterized the responses of the fabricated sample with respect to refractive index change of glucose solution and temperature change of DI water. Figure 5a shows the reflection spectra of the nanoprobe at different glucose concentrations (i.e., different refractive indices) at room temperature (T_0_ = 25.9 °C). Both resonance dips linearly redshift as the refractive index is increased (Fig. 5b,c). However, the shift of Dip Y is much larger than that of Dip X since the mode corresponding to Dip Y is distributed mainly in the ambient solution side but that of Dip X in the silica side. The measured ${s}_{{\rm{\Delta }}n}^{X}$ and ${s}_{{\rm{\Delta }}n}^{Y}$ are 29.1 nm/RIU and 1150.8 nm/RIU, respectively, which compare well with the simulation results.

The temperature sensitivities of the nanoprobe were measured at different temperatures of DI waters (n_0_ = 1.3329 at room temperature). Figure 5d shows the reflection spectra at different temperatures. As the temperature increases, Dip X redshifts linearly (sensitivity of 0.0059 nm/°C) (Fig. 5e) but Dip Y blueshifts (sensitivity of −0.2287 nm/°C) with weak nonlinearity (Fig. 5f). Note that these dip shifts result from the thermo-optic effects of both silica and DI water. Therefore, to obtain the temperature sensitivities solely due to the thermo-optic effects of silica (i.e., ${s}_{{\rm{\Delta }}T}^{X}$ and ${s}_{{\rm{\Delta }}T}^{Y}$), the shifts due to the thermo-optic effect of DI water should be removed.

Note that silica has a positive thermo-optic coefficient (1 × 10^−5^/°C), while the refractive index of DI water decreases nonlinearly with temperature. Considering the simulated sensitivity of Dip Y (redshifts with increased refractive index) to the refractive index change of silica is only 5.7 nm/RIU, the corresponding ${s}_{{\rm{\Delta }}T}^{Y}$ (i.e., 5.7 × 10^−5^ nm/°C) is negligible. Therefore, the observed blueshift of Dip Y can be assumed to be solely due to the thermo-optic effect of DI water and the ${s}_{{\rm{\Delta }}T}^{Y}$ can be assumed to be 0 nm/°C.

The sensitivity ${s}_{\Delta T}^{X}$ solely due to the thermo-optic effect of silica can be obtained from the measured Dips X and Y shifts as follows. First, we obtain the temperature dependence of the refractive index of DI water (Fig. 5h) from the ${s}_{{\rm{\Delta }}n}^{Y}$ (Fig. 5c) and the temperature-dependent Dip Y shifts (Fig. 5f). Second, Dip X shifts due to the thermo-optic effect of DI water (i.e., refractive index change of the DI water) are obtained using the ${s}_{{\rm{\Delta }}n}^{X}$ (Fig. 5b) and the temperature-dependent refractive index of DI water (Fig. 5h), which has a negative sensitivity of −0.0058 nm/°C. Finally, Dip X shifts due to the thermo-optic effect of silica (∆Dip X_silica_) (Fig. 5g) are obtained by subtracting the Dip X shifts due to the thermo-optic effect of DI water from the overall temperature-dependent Dip X shifts (Fig. 5e). The measured ${s}_{{\rm{\Delta }}T}^{X}$ is 0.0117 nm/°C (i.e., 1170.0 nm/RIU), which agrees well with the simulations.

The sensitivity matrix of the fabricated nanoprobe can therefore be expressed as

5 $$S=(\begin{array}{c}\begin{array}{cc}29.1nm/RIU & 0.0117nm/^\circ C\end{array}\\ \begin{array}{cc}1150.8nm/RIU & 0nm/^\circ C\end{array}\end{array}).$$

The experiment characterizations of the fabricated nanoprobe demonstrate that the two resonance dips have distinctive responses to the refractive index change of ambient solution and the temperature change. With the obtained sensitivity matrix S and the references T_0_ = 25.9 °C and n_0_ = 1.3329, the nanoprobe can be used to measure the refractive index and temperature of unknown solutions simultaneously in a single measurement.

## Conclusions

We developed a lab-on-fiber multiparameter nanoprobe for simultaneous sensing of solution refractive index and temperature. The nanoprobe entailed a plasmonic crystal cavity enhanced by DBRs on the end-face of a single-mode optical fiber. By tailoring the grating periods of the plasmonic crystal cavity and DBRs, the two spatially separated high-Q RA-SPP resonance modes were designed to be within a 50 nm spectral range at 1550 nm and had distinctive sensitivities to the solution refractive index and temperature changes. The designed nanoprobe was fabricated with direct FIB milling of the Au film deposited on the fiber end-face. With the fabricated nanoprobe two RA-SPP resonance dips in its reflection spectrum were clearly observed at the 45°-angled linear polarization. Dip X was at 1535.25 nm with a Q-factor of 198 and Dip Y was at 1583.12 nm with a Q-factor of 41. Furthermore, the two resonances were demonstrated to have distinctive responses to the changes of refractive index and temperature, which enables the nanoprobe to measure both parameters simultaneously. Note that the nanoprobe was designed to operate in the DI water environment. However, it can be used in various mediums (e.g., gases and organic solvents), which have significantly different refractive indices from DI water. In these cases, the grating periods of the nanoprobe would need to be adjusted to position the two resonance dips in the C + L band. Therefore, the proposed plasmonic nanoprobe is expected to serve as a promising lab-on-fiber platform for multifunctional and multiparameter analysis.

## Methods

### Optical simulation

The optical simulations were carried out by using FDTD software (Lumerical FDTD Solutions). For the unit cell 1D Au grating model (Fig. 2a), Bloch and perfectly matched layer (PML) boundary conditions were used for the x and z boundaries, respectively. A TM plane wave was used as incident light. For the unit cell 2D Au grating model (Fig. S1a), the Bloch boundary condition was used for the x and y boundaries and PML was used for the z boundaries. A plane wave with 45°-angled linear polarization was used as incident light. For the on-fiber plasmonic crystal cavity model (Fig. 2d), PML was used for all the boundaries. The diameters of the fiber core and cladding were 9 µm and 125 µm, respectively. The cavity lengths were 14.42 µm in the x direction and 14.76 µm in the y direction. A fiber guided mode was used for incident light. For both models the refractive index of Au was adapted from the materials database of the FDTD software^36^, and the refractive indices of the core and cladding were 1.450 and 1.445, respectively. The ambient solution was DI water, whose refractive index was 1.333.

### Nanofabrication

The multiparameter nanoprobe was fabricated on the end face of a single-mode optical fiber (SMF-28, corning). The optical fiber was cleaved so that the fiber end-face was perpendicular to the longitudinal direction. The cleaved fiber tip was cleaned with acetone, isopropyl alcohol, and DI water, followed by nitrogen dry. A thin Au/Ti (60 nm/3 nm) film was deposited on the fiber end-face using electron-beam evaporation (Denton Vacuum Explorer 14, Denton Vacuum). Note that the 3 nm Ti layer was used to enhance the adhesion between the Au film and the fiber end-face. To verify the Au/Ti film thickness, a glass substrate was loaded near the optical fiber end-face and deposited with the Au/Ti film. The thickness of the Au/Ti film on the glass substrate was measured by using a profilometer (Alpha Step 200, Tencor). The deposited Au/Ti film was patterned to create the plasmonic crystal cavity by FIB (GAIA3, Tescan) milling.

### Characterization

Four different concentrations of glucose solutions were used for refractive index sensing. The glucose solutions were prepared by dissolving glucose powder (Dextrose, Sigma Aldrich) into room temperature DI water. The refractive indices of the glucose solutions were measured using a refractometer (Digital Brix/RI-Chek, Reichert) with an accuracy of 2 × 10^−4^. The measured refractive indices were 1.3329, 1.3424, 1.3492, and 1.3565 for 0%, 6%, 12%, and 18% concentration solutions, respectively, which showed a linear relation of 0.0013 RIU/%. Temperature sensing was carried out in DI water. The DI water was heated up to 80 °C on the hot plate. The DI water temperature was measured while it was cooled down to room temperature. Throughout the measurement it was stirred with a magnetic bar to achieve a uniform temperature distribution across the beaker. A Type-K thermocouple (CO1, Omega) with a signal processing unit (CN77333, Omega) was used as a reference temperature sensor, which has an accuracy of 0.4 °C.

## Supplementary information

Supplementary information

## Data Availability

The dataset generated or analyzed during the current study are available from the corresponding author on reasonable request.
