# Supplementary material for: Lab-on-Fiber Nanoprobe with Dual High-Q Rayleigh Anomaly-Surface Plasmon Polariton Resonances for Multiparameter Sensing
Source: Sci Rep. 2019 Feb 13;9:1922. doi: 10.1038/s41598-018-38113-1 (PMC6374433; doi:10.1038/s41598-018-38113-1)
Supplement: Supplementary file 1 — Supplementary information [file 41598_2018_38113_MOESM1_ESM.pdf]

Supplementary information:

Lab-on-Fiber Nanoprobe with Dual High-Q Rayleigh Anomaly-Surface Plasmon Polariton Resonances for Multiparameter Sensing

Hyun-Tae Kim and Miao Yu*

Department of Mechanical Engineering, University of Maryland, College Park, Maryland 20742, USA
*mmyu@umd.edu

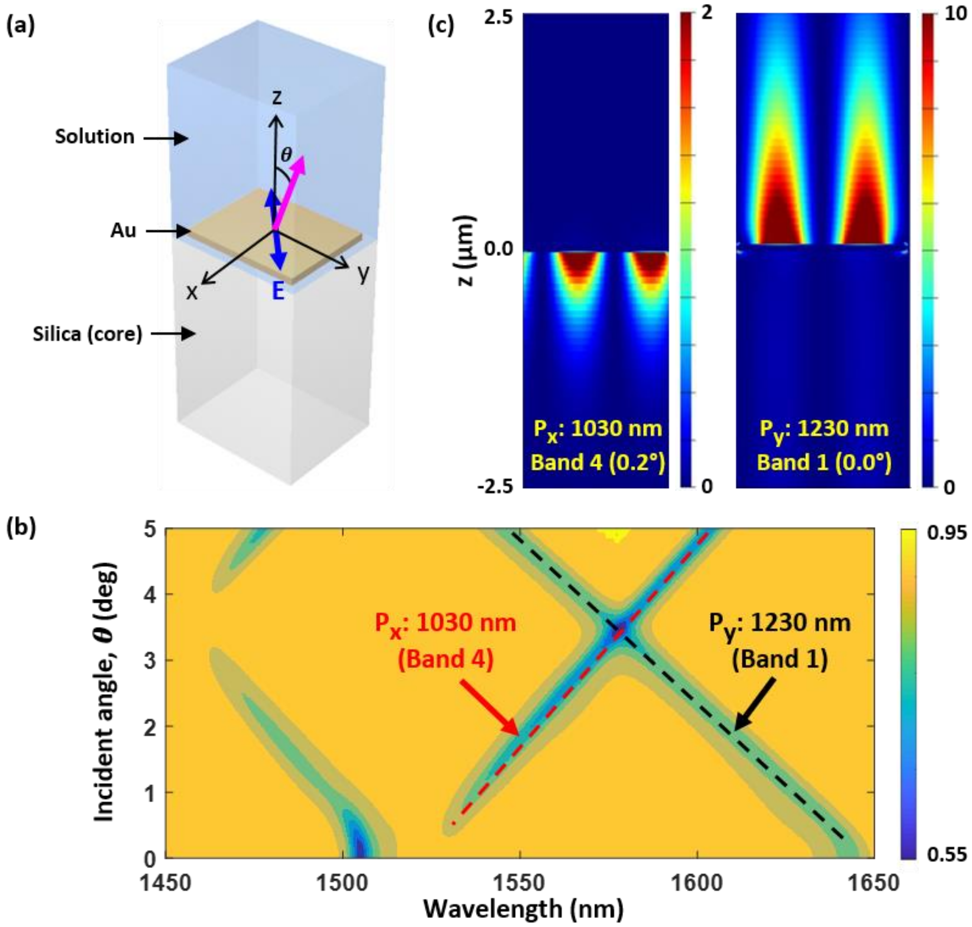

Figure S1. 3D simulations of a photonic crystal cavity with a 2D rectangular Au grating. (a) Schematic of a unit cell of the 2D grating with an incident plane wave (45˚-angled linear polarization). (b) Simulated reflection spectra of the infinite 2D Au grating with P_x = 1030 nm and P_y = 1230 nm at different incident angles obtained with 3D FDTD simulations. (c) Simulated band edge mode profiles of the 2D grating at near normal incident angles.

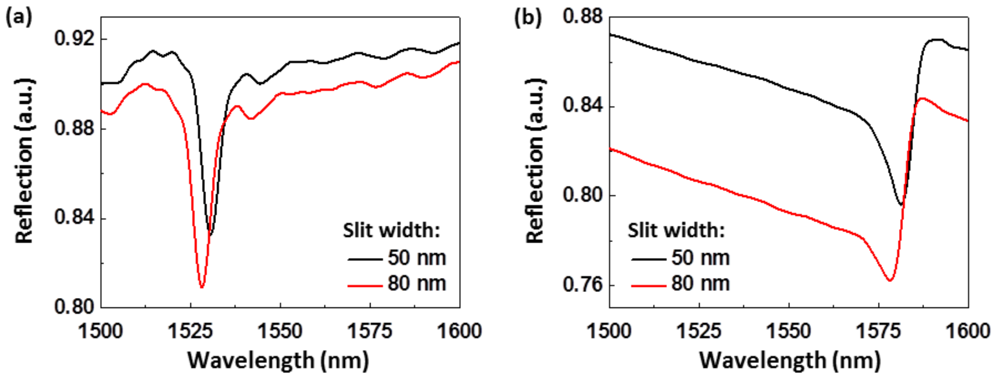

Figure S2. Simulated reflection spectra of the 1D on-fiber plasmonic crystal cavities with different slit widths: cavities with a core grating period of (a) 1030 nm (DBR: 525 nm, gap: 900 nm) and (b) 1230 nm (DBR: 590 nm, gap: 550 nm).
